# The Risk of Familial Mediterranean Fever in *MEFV* Heterozygotes: A Statistical Approach

**DOI:** 10.1371/journal.pone.0068431

**Published:** 2013-07-03

**Authors:** Isabelle Jéru, Véronique Hentgen, Emmanuelle Cochet, Philippe Duquesnoy, Gaëlle Le Borgne, Emmanuel Grimprel, Katia Stankovic Stojanovic, Sonia Karabina, Gilles Grateau, Serge Amselem

**Affiliations:** 1 UMR_S933, Institut National de la Santé et de la Recherche Médicale (INSERM), Paris, France; 2 UMR_S933, Université Pierre et Marie Curie-Paris6 (UPMC), Paris, France; 3 Service de Génétique et d'Embryologie médicales, Hôpital Trousseau, Assistance Publique-Hôpitaux de Paris (AP-HP), Paris, France; 4 Département hospitalo-universitaire Inflammation – Immunopathology – Biotherapy, UPMC, AP-HP, INSERM and Centre National de la Recherche Scientifique (CNRS), Paris, France; 5 Centre de Référence des Maladies AutoInflammatoires (CeRéMAI), Centre Hospitalier de Versailles, Le Chesnay, France; 6 Service de Pédiatrie Générale, Hôpital Trousseau, AP-HP, Paris, France; 7 Centre de référence Amyloses d'origine inflammatoire et Fièvre méditerranéenne familiale, Hôpital Tenon, AP-HP, Paris, France; University of Thessaly, Faculty of Medicine, Greece

## Abstract

**Background:**

Familial Mediterranean fever (FMF) is an autosomal recessive autoinflammatory disorder due to *MEFV* mutations and one of the most frequent Mediterranean genetic diseases. The observation of many heterozygous patients in whom a second mutated allele was excluded led to the proposal that heterozygosity could be causal. However, heterozygosity might be coincidental in many patients due to the very high rate of mutations in Mediterranean populations.

**Objective:**

To better delineate the pathogenicity of heterozygosity in order to improve genetic counselling and disease management.

**Methods:**

Complementary statistical approaches were used: estimation of FMF prevalence at population levels, genotype comparison in siblings from 63 familial forms, and genotype study in 557 patients from four Mediterranean populations.

**Results:**

At the population level, we did not observe any contribution of heterozygosity to disease prevalence. In affected siblings of patients carrying two *MEFV* mutations, 92% carry two mutated alleles, whereas 4% are heterozygous with typical FMF diagnosis. We demonstrated statistically that patients are more likely to be heterozygous than healthy individuals, as shown by the higher ratio heterozygous carriers/non carriers in patients (p<10^−7^–p<0.003). The risk for heterozygotes to develop FMF was estimated between 2.1×10^−3^ and 5.8×10^−3^ and the relative risk, as compared to non carriers, between 6.3 and 8.1.

**Conclusions:**

This is the first statistical demonstration that heterozygosity is not responsible for classical Mendelian FMF per se, but constitutes a susceptibility factor for clinically-similar multifactorial forms of the disease. We also provide a first estimate of the risk for heterozygotes to develop FMF.

## Introduction

Familial Mediterranean fever (FMF) is part of the expanding family of autoinflammatory disorders and one of the most frequent genetic disorders in the Mediterranean basin, especially in Turkish, Arab, Jewish and Armenian populations. FMF diagnosis remains often difficult due to the lack of pathognomonic signs. The recurrent episodes of fever and systemic inflammation, which last a few days and commonly appear during infancy, are accompanied by peritonitis, arthritis, pleurisy, and skin manifestations. Systemic AA-amyloidosis, which may progress to terminal renal failure, represents the main complication [1]. Patients are usually treated by life-long colchicine administration. The autosomal recessive transmission of FMF was established more than 15 years ago by several means: familial studies [2]–[4], linkage analysis leading to the identification of the disease causing gene (*MEFV*) [5]–[6], haplotype comparisons, and identification of two *MEFV* mutations in many patients.

*MEFV*, which encodes a protein called pyrin, is primarily expressed in cells from the myelomonocytic lineage and in synovial fibroblasts [7]–[8]. Although there has been a lot of controversy about the precise function of pyrin, this protein seems to regulate the inflammatory response through its action on IL-1β signalling pathway. Most *MEFV* sequence variations identified to date correspond to missense changes (Infevers website, http://fmf.igh.cnrs.fr/ISSAID/infevers/). Their deleterious effect is difficult to establish since the gene is not well conserved throughout evolution and since there is no validated routine functional test to assess their pathogenicity. A subset of *MEFV* mutations has been shown to explain a majority of cases in Mediterranean populations [9]. The risk of a false negative is extremely low when the few most frequent mutations are searched for [10]–[18], so that routine molecular diagnosis usually screens for a limited number of mutations. In those at-risk populations, the frequency of heterozygous carriers is particularly high (up to 20%) so that a pseudo-dominant mode of inheritance has been reported in some families. Noteworthy, in FMF patients who are not from Mediterranean populations, *MEFV* mutations are extremely rare, suggesting that other genes might be responsible for FMF phenotypes [19]–[20]. In addition, a number of patients from Mediterranean ancestry also remain genetically unexplained, that is, they do not carry two mutated *MEFV* alleles.

Recently, the observation of such Mediterranean patients presenting with typical FMF manifestations, usually responding well to colchicine, and carrying a single heterozygous *MEFV* mutation, led to the idea that FMF might appear in heterozygotes. There was no direct proof to support this hypothesis since no cellular mechanism or molecular explanation was proposed to confirm this idea. The causality of heterozygosity was proposed after thorough search and exclusion of the presence of a second mutated allele. Indeed, several teams screened the entire coding [10]–[13], [17]–[18], or the whole genomic *MEFV* sequence [16]. Analysis of *MEFV* RNA in patients excluded transcript size abnormalities, and identification of SNP after cDNA sequencing ruled out allele silencing [15]–[16]. Multiplex ligation probe amplification also failed to reveal any copy number variation [14]–[15]. A population genetics-based study assessing fitness with Hardy-Weinberg equilibrium demonstrated that FMF manifestations are unrelated to *MEFV* in most genetically-unexplained patients from at-risk populations [13]. Consistently, the presence of different haplotypes for the second allele in affected siblings carrying a single *MEFV* mutation ruled out the presence of an unidentified mutation [15]–[16], [21]. Finally, genotype studies in a few families with an autosomal dominant disease mode of inheritance contributed to the idea that heterozygosity might be responsible for the disease [12], [15], [21]–[23].

However, a crucial issue for genetic counselling remains unresolved: some of these patients presenting manifestations evocative of FMF might happen to be heterozygotes coincidentally due to the high frequency of *MEFV* variants in at-risk populations. Lacking an alternative explanation, it might seem tempting to consider heterozygosity as causal; however, it could be risky to take it as a general rule since this might lead to misdiagnosis. We undertook this study to better delineate the pathogenicity of heterozygosity by means of a number of complementary statistically-based approaches, applied to very large study groups comprising a total of 557 patients and 63 familial forms.

## Patients and Methods

### Patients

This study was approved by the Comité de Protection des Personnes Ile-de-France 5, Paris, France. In this retrospective study, we included 557 unrelated consecutive patients who were clinically diagnosed as having FMF and who met the established set of Tel Hashomer's diagnostic criteria [24]. They were all referred to our National Reference Centres for molecular diagnosis of their autoinflammatory syndromes. Informed written consent was given by all individuals or, in the case of children, by their legal guardians. All the patients included in this study had two parents originating from one of the most affected populations (Armenian, Turkish, North African Sephardic Jewish, Arab). Clinical features, origins, and familial history were recorded through a standardized form. Among the patients included, 63 carried 2 mutated *MEFV* alleles and were part of multiplex families, which were used to test whether affected siblings carried one or two mutated *MEFV* alleles.

### *MEFV* analysis

gDNA was extracted from peripheral blood leukocytes (FlexiGene, Qiagen). Molecular analyses were performed within the framework of routine genetic testing. All unambiguous *MEFV* mutations were systematically searched for, as well as the E148Q sequence variation since it is quite frequent and its deleterious effect is much debated. Rare variations of unknown pathogenicity were not considered as disease-causing mutations, especially since their low frequencies did not affect statistical calculations.

### Statistical analyses

Comparisons were performed using Pearson's chi-square tests or Fisher's exact tests. Differences were considered statistically significant at p≤0.05.

## Results

### Study of FMF prevalence in at-risk populations

We first evaluated at the population level the contribution of heterozygosity to the prevalence of the disease. To this end, we compared the FMF prevalence estimated on the basis of clinical reports to the prevalence calculated from the frequency of the mutated *MEFV* alleles. If heterozygosity played a significant role in FMF occurrence, the observed prevalence (P) should stand in between q^2^<P<2pq+q^2^ (q: frequency of mutated *MEFV* alleles; p: frequency of normal *MEFV* alleles; q^2^: frequency of individuals carrying two mutated alleles; 2pq: frequency of heterozygotes). Notably, the q value depends on what is considered as a disease-causing mutation. The problem is particularly true for the debated E148Q sequence variation, since it is quite frequent in at-risk populations; other sequence variations whose deleterious effect is not established are rare and their respective frequencies did not significantly affect calculations. We found two estimations of FMF prevalence based on clinical reports: one in Sephardic Jews [25] and the other in Turks [26]. Table 1 presents the comparative analysis of the observed and calculated prevalence in these populations. First of all, these data did not reveal any detectable contribution of heterozygosity to FMF prevalence at the population level. Indeed, the prevalence calculated according to a model of autosomal recessive transmission (P = q^2^) is already slightly more elevated than the observed one, suggesting that the disease is underdiagnosed or that certain mutations have reduced penetrance. Notably, the calculated prevalence matches the observed one, only if E148Q is considered as a polymorphism or as a sequence variation with very low disease penetrance.

**Table 1:** Comparison of the FMF prevalence estimated on clinical reports to the prevalence calculated from the frequency of mutated *MEFV* alleles.

Population	FMF prevalence				
	Estimated on the basis of clinical reports	Calculated a,b on the assumption that P = q^2^		Calculated a,b on the assumption that P = 2pq+q^2^	
		Considering E148Q as a polymorphism	Considering E148Q as a disease-causing mutation	Considering E148Q as a polymorphism	Considering E148Q as a disease-causing mutation
**Turkish**	0.001 [26]	0.003	0.01	0.1	0.2
**Sephardic Jewish**	0.001–0.004 [25]	0.005	0.02	0.1	0.2

P: FMF prevalence; q: frequency of mutated *MEFV* allele; p: frequency of normal *MEFV* allele.

a,b Calculations were made using q frequencies estimated previously in the Turkish (q = 0.05) [37] and Sephardic Jewish (q = 0.09) populations [38].

### *MEFV* genotyping in affected siblings of patients with clinical FMF and two mutated alleles

**Figure 1:**
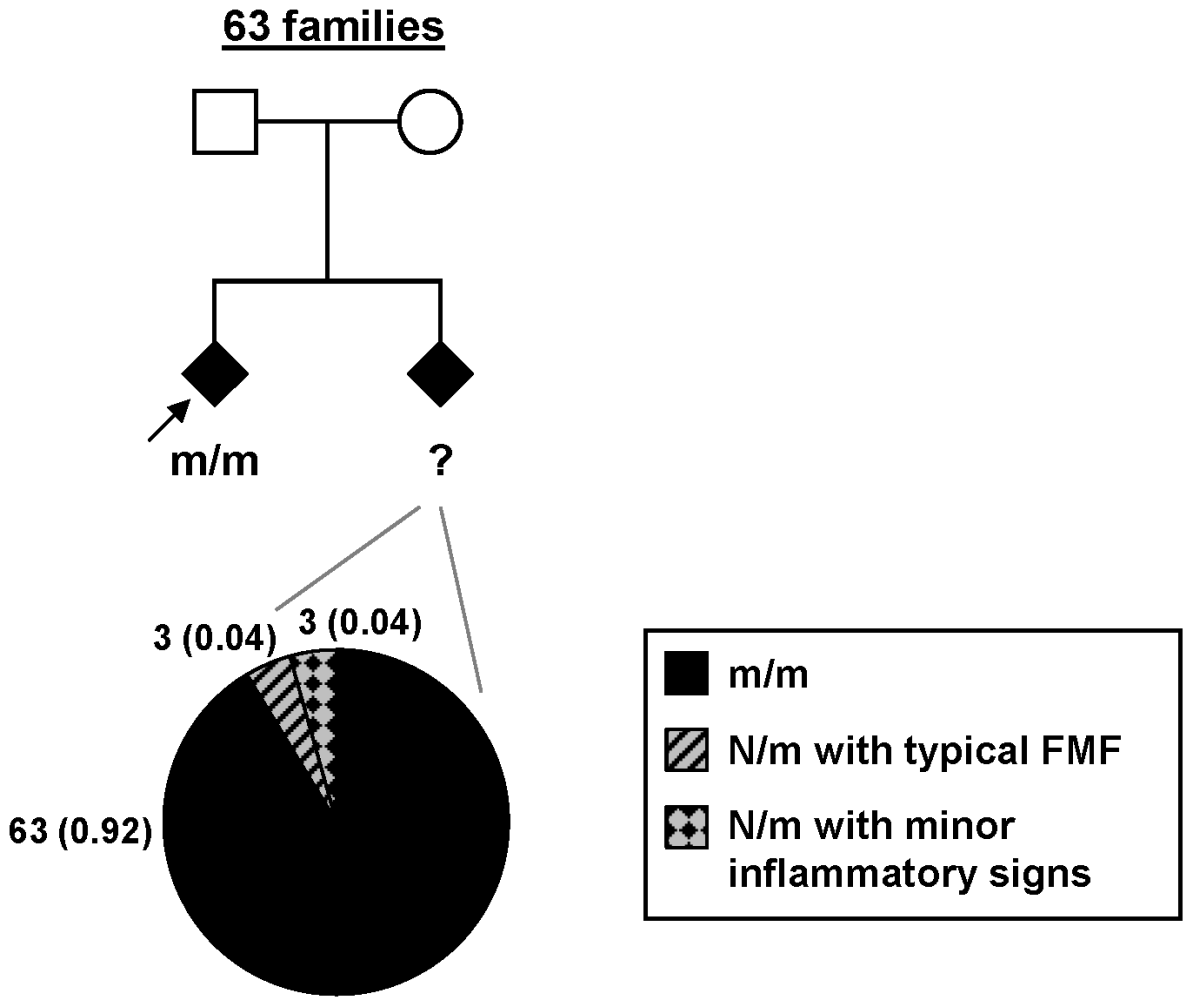
*MEFV* genotype distribution in affected siblings of FMF probands. The upper part of the figure gives a schematic representation of the FMF familial forms included in this analysis: each proband presented a clinical diagnosis of FMF, carried two *MEFV* mutations and had at least one affected sibling. The lower part of the figure displays the *MEFV* genotype distribution in affected siblings. Numbers in each genotype class are indicated by labels next to each sector; numbers in brackets correspond to percentages. m: mutated *MEFV* allele; N: normal *MEFV* allele.

We evaluated the percentage of heterozygotes in affected siblings of FMF probands in whom the diagnosis was genetically confirmed (i.e. two mutated alleles) (Figure 1). All families meeting these criteria and available in our database (n = 63) were included. In these typical familial forms, the mutations identified were F479L, M680I, M694V, M694I, V726A and R761H. Taken together the 63 index cases had 69 siblings with FMF manifestations. Among them, 63 (92%) carried two mutated alleles and only 6 were heterozygotes (Figure 1). In addition, the diagnosis of FMF was not retained in three affected siblings (4%) that were heterozygous carriers: two patients had one isolated sign; one individual had only two episodes of abdominal pain and arthralgia. As for the three other heterozygotes (4%), they had clinical FMF, but only one with the same severity as his sibling. It is also important to note that, even if some of them might develop disease manifestations later in life, 16 unaffected heterozygous carriers were also present in those siblings. All these data strongly suggest that heterozygosity plays only a minor role in the typical familial forms of FMF.

### Frequency of heterozygotes among FMF patients

**Figure 2:**
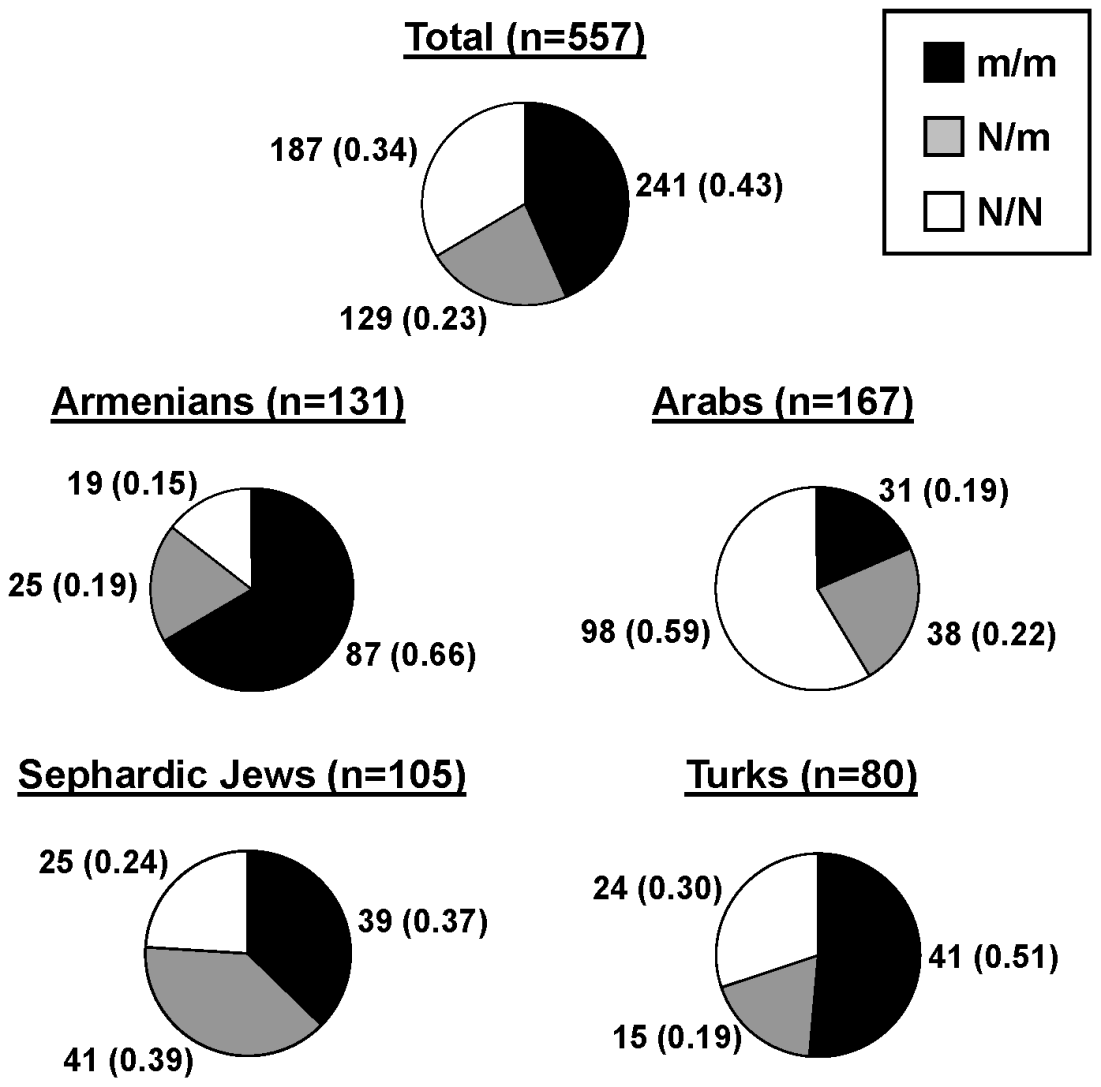
*MEFV* genotype distributions in FMF patients from at-risk origin and meeting established clinical criteria. All included patients (n = 557) were unrelated, had a clinical diagnosis of FMF, met Tel Hashomer's criteria and had parents originating from one of the most at-risk populations. The genotype distribution in all patients fulfilling these inclusion criteria is presented at the top. Detailed genotype distributions according to the origin of patients are displayed below. n indicates the number of patients in each group. Numbers in brackets correspond to percentages. The total number of patients is higher than the sum in each at-risk population, since a few patients had parents from two different at-risk origins. m: mutated *MEFV* allele; N: normal *MEFV* allele.

We then studied in detail the *MEFV* genotypes in FMF patients referred for molecular diagnosis to our National Reference Centres for autoinflammatory disorders. The first hurdle encountered when one attempts to address the question of the causality of *MEFV* heterozygous mutations is the choice of inclusion criteria for the patients, which are not always mentioned in studies related to this issue. In order to focus on the most typical cases, we included consecutive unrelated patients who were clinically diagnosed as having FMF, meeting the established set of Tel-Hashomer's criteria [24], and with two parents from one of the most affected populations (Armenian, Turkish, North African Sephardic Jewish, Arab). 557 independent patients fulfilled these criteria, among whom 129 (23%) carried a single *MEFV* mutation, and 187 (34%) had no *MEFV* molecular defect. Detailed genotype distributions showing the percentages of patients carrying 0, 1 or 2 mutated alleles in each at-risk population are presented in Figure 2 and indicate that a great proportion of patients remains genetically unexplained with a high proportion of heterozygotes. Notably, the spectrum of *MEFV* mutations identified among heterozygotes was similar to that present in patients carrying two mutated alleles (data not shown).

A first explanation to the high number of genetically-unexplained patients could very well be clinical misdiagnosis with a disease mimicking FMF and unrelated to *MEFV*. According to this hypothesis, we could expect the ratio [heterozygous carriers/non carriers] (R) at the *MEFV* locus to be similar in genetically-unexplained patients and in the general population from the same origin. In genetically-unexplained Armenian patients (n = 44) (Figure 2), the R ratio was very high (25/19 = 1.32). Previous studies evaluated the frequency of mutated *MEFV* alleles in the Armenian population (q∼0.07) [4], [27], allowing us to estimate R at 2pq/p^2^ = 0.15. We then deduced the expected number of heterozygous carriers (n = 5.74) and non carriers (n = 38.26) in a group of 44 individuals (same size as the study group) from the general population. Comparison, using chi-square tests, of the R ratios observed among genetically-unexplained patients and those expected in the general population revealed a significant difference (p<2×10^−5^) (Table 2). Consistent with these results, we also observed significant differences in other populations: Sephardic Jews (1.64 vs 0.2; p<10^−7^), Arabs (0.39 vs 0.08; p<10^−5^), Turks (0.63 vs 0.1; p<3×10^−3^) (Table 2). This clearly demonstrates that there is a marked excess of heterozygotes among genetically-unexplained patients.

**Table 2:** Comparison of the ratio [heterozygous carriers/non carriers] (R = 2pq/p^2^) observed in genetically-unexplained FMF patients and expected in several origin-matched populations.

	Armenians	North African Sephardic Jews	Arabs	Turks
**R observed in genetically-unexplained patients**	25/19 = 1.32	41/25 = 1.64	38/98 = 0.39	15/24 = 0.63
**R expected in the general population** a	5.74/38.26 = 0.15	11/55 = 0.2	10.07/125.93 = 0.08	3.86/35.14 = 0.1
**p-values**	<2×10^−5^	<10^−7^	<10^−5^	<3×10^−3^

a q frequencies used to calculate R ratios in general control populations were taken from previous reports: q = 0.07 for Armenians [4], [27], q = 0.09 for North African Sephardic Jews [38], q = 0.04 for Arabs [39], and q = 0.05 for Turk [37].

### Estimation of the risk and relative risk for heterozygotes to develop FMF

**Figure 3:**
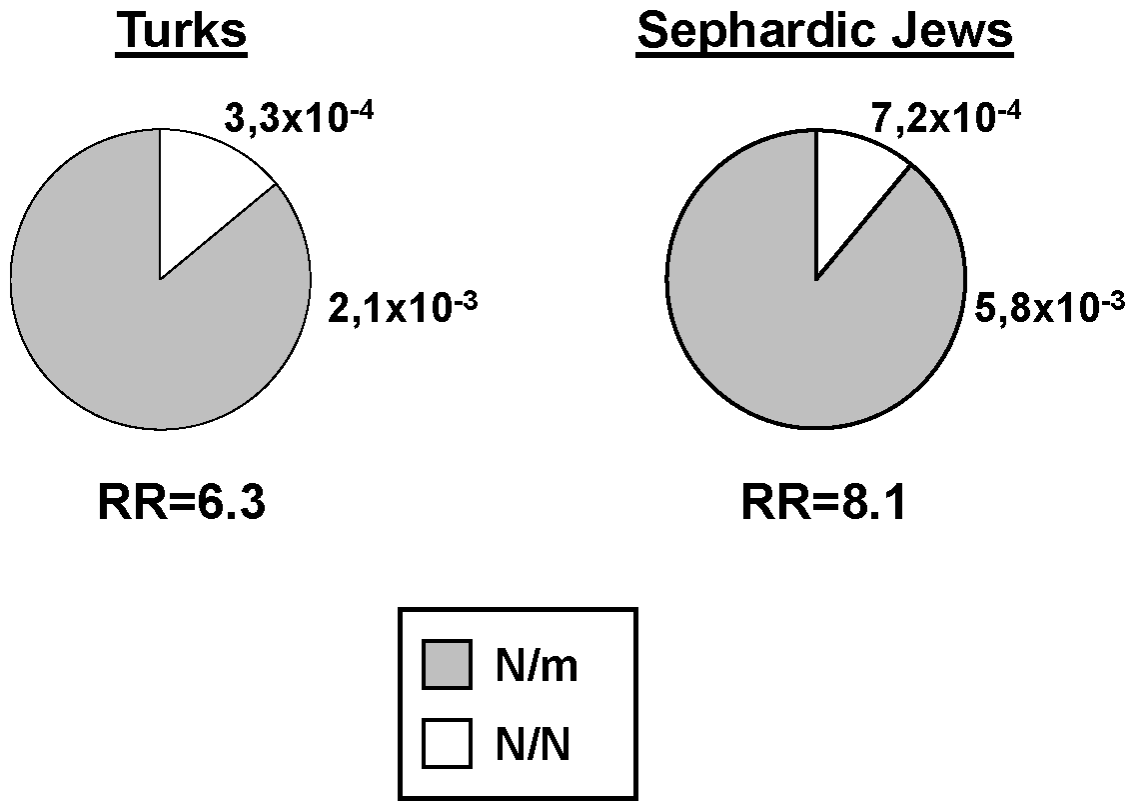
Risk and relative risk for heterozygotes to develop FMF. Considering the prevalence of FMF reported in Turks and Sephardic Jews [25]–[26], as well as the distribution of *MEFV* genotypes in affected and healthy individuals, we could make a rough estimate of the risk and relative risk for heterozygotes to develop FMF, as compared to healthy individuals. Risks are indicated by labels next to each sector, relative risks are displayed below each genotype distribution. m: mutated *MEFV* allele; N: normal *MEFV* allele.

Although it is a crucial point for genetic counselling, the question of the risk for heterozygotes to develop FMF manifestations remains largely unanswered. Calculation of the increase in disease risk associated with heterozygous mutations is a highly difficult task since it involves the study of very large cohorts taken at random in the general population. Nevertheless, knowledge of the FMF prevalence observed in a given population, combined with the distributions of *MEFV* genotypes among patients and healthy individuals can provide a rough estimate of the risk and relative risk (RR) for heterozygotes to develop FMF, as compared to individuals carrying no *MEFV* mutations. As an example, we detail the estimation of the RR in Turks. The prevalence of FMF in Turkey is 0.001 [26], so that in a group of 10^5^ individuals taken at random, there should be 100 patients and 99,900 healthy subjects. Among patients, 51 individuals (51%) are expected to carry two mutated alleles, 19 should be heterozygous and 30 should carry no mutation, according to our data displayed in Figure 2. Among healthy subjects, 9,082 individuals should be heterozygous and 90,818 should carry no mutation, according to the R ratio presented in Table 2. Therefore, the risk for heterozygotes to develop clinical manifestations is 2.1×10^−3^ (19/(19+9,082)). In a similar way, we could estimate the risk for heterozygotes to develop FMF in Sephardic Jews at 5.8×10^−3^. The corresponding relative risks as compared to individuals carrying no *MEFV* mutation range from 6.3 to 8.1 (Figure 3).

## Discussion

The putative pathogenicity of heterozygosity in FMF is usually deduced from the fact that, in spite of complete *MEFV* screening, a number of patients with typical manifestations carry a single mutated allele. However, this does not constitute any direct proof since *MEFV* mutations might be present in some patients coincidentally, due to their very high frequencies in at-risk populations. The current study is original in that it takes advantage of statistical approaches to better delineate the pathogenicity of heterozygosity in FMF. Estimations of the disease prevalence at the population level, observations in typical familial forms, and thorough investigation of several hundreds of patients allow us to provide the first statistical demonstration that heterozygosity is not responsible for the classical Mendelian FMF per se, but constitutes a susceptibility factor for clinically-similar multifactorial forms of the disease. We provide a first estimate of the risk and relative risk for heterozygotes to develop FMF and demonstrate that the disease is present only in a small subset of heterozygotes. The fact that we made similar observations in patients from different origins reinforces our conclusions.

Our data show no clear contribution of heterozygosity to the disease prevalence in population genetics-based studies. Indeed, if we consider that two mutations are necessary for disease occurrence, the FMF prevalence calculated from the frequency of mutated *MEFV* alleles is already slightly higher than the one observed. Consistently, we observed in typical familial forms in which FMF probands were found to carry two unambiguous *MEFV* mutations that their affected siblings also carry two mutated alleles in the vast majority of cases (92%). In the few exceptions to the rule (FMF siblings carrying a single mutated allele), FMF diagnosis should be evaluated cautiously in order to distinguish FMF from inflammatory manifestations evocative of FMF in the context of a familial history.

However, the ratio heterozygous carriers/non carriers is far higher in genetically-unexplained patients than in the general population from the same origin, showing that recruitment of patients presenting with FMF manifestations favours the recruitment of heterozygotes. These apparently conflicting data can be easily reconciled if we consider that a single heterozygous mutation is not sufficient to trigger FMF, but constitutes a susceptibility factor for the disease, which appears in a small subset of individuals when combined with additional molecular defects and/or environmental factors. The current study thereby provides the first statistical demonstration that heterozygosity for a single *MEFV* mutation constitutes a susceptibility factor for FMF and that some heterozygous patients do not carry a mutated *MEFV* allele coincidentally. Notably, several additional clues argue for a role of heterozygosity in disease development: (i) many heterozygotes have mild elevation of acute phase reactants such as CRP or SAA [28]–[29]; (ii) heterozygous parents of FMF children have more inflammatory manifestations in their medical history than controls [30]; (iii) *MEFV* expression is increased and similar in patients carrying a single or two mutated allele(s) [16].

Are we then able to clinically distinguish the classical Mendelian FMF and the complex forms of the disease? Several previous reports indicate that symptoms might be milder or less typical in heterozygotes [15]–[17], [21], [28], [31]–[33]. A recent report from Federici et al. also showed a decrease in the frequency of the most typical FMF manifestations from patients carrying two high penetrance mutations towards patients with a single low penetrance mutation [34]. Peculiarities in response to treatment have also been reported, such as unresponsiveness [16], [21] or sustained remission after colchicine withdrawal [32]–[33]. However, there is today no obvious element or severity score to clearly differentiate between the different forms, so that FMF clinical definition remains a challenge.

A crucial issue for clinicians and patients is the risk for heterozygotes to develop FMF manifestations. We evaluated this risk between 2.1×10^−3^ and 5.8×10^−3^ and the relative risk, as compared to individuals carrying no mutation, between 6.3 and 8.1. These data correspond to estimations generated from knowledge of the FMF prevalence and distribution of *MEFV* genotypes in at-risk populations and should be confirmed in dedicated prospective studies. Nevertheless, these observations are in accordance with our previous results showing that the contribution of heterozygosity at the population level or in most typical familial forms is small. Consequently, although the exact risk for heterozygotes to experience mild inflammatory signs might be difficult to evaluate precisely, the percentage of heterozygotes from the general population presenting typical FMF manifestations remains very low. As for the risk of recurrence in a given family, it is difficult to assess. Indeed, the study of the frequency of FMF in the relatives of a heterozygous proband raises several obvious problems. First of all, healthy individuals have a priori no reason to come and see a clinician to be genotyped. Secondly, considering that FMF usually appears during childhood or teenage years, the risk of recurrence could only be evaluated in a cohort of adults, thereby greatly limiting the number of candidates.

One major aim of this study was to avoid wrong interpretation of heterozygosity, which might lead to set false positive diagnoses and to neglect genetic heterogeneity in autoinflammatory disorders evocative of FMF. The data presented herein bring to light two messages that are decisive for genetic counselling: (i) heterozygosity should not be considered as sufficient to establish a molecular diagnosis of FMF; (ii) heterozygosity is a susceptibility factor for FMF, which appears in a very small subset of individuals. Consequently, clinical judgment remains crucial in establishing the diagnosis. Detection of a single heterozygous mutation, in the presence of clear clinical symptoms, appears to be sufficient for a colchicine trial [32]–[33]. It is also important to keep in mind that most FMF patients who do not belong to at-risk populations do not carry any *MEFV* mutations. In addition, a subset of FMF patients from at-risk origins does not carry any mutated *MEFV* allele.

At the present time, we have no clue as to the allelic architecture in the genetically-unexplained FMF forms. Do we have to deal with one or few genetic variants of large effects or with a number of common variants, which individually or in combination confer small increments in risk? What is the environmental contribution? Is there any influence of epigenetic factors, whose involvement in autoinflammatory diseases still remains elusive [35]? The term “missing heritability”, usually used to describe the gap between predictive transmission models of complex traits and statistical explanatory power of susceptibility genes identified by genome-wide association studies (GWAS) [36], could also apply to this particular situation in which a small subset of individuals carrying a single *MEFV* mutation develops FMF manifestations, in the presence of so-far unidentified factors. Identification of such factors and characterization of their interaction with *MEFV* is a challenging issue. Limited size of homogeneous groups of patients, imprecise phenotyping, and difficulty in accounting for shared environment among relatives, would indeed constitute major limitations in the design of GWAS. However, the current study represents an additional step to throw a bridge across the gap separating the Mendelian and the multifactorial forms of FMF.

## References

[1] Zemer D, Pras M, Sohar E, Modan M, Cabili S, et al (1986) Colchicine in the prevention and treatment of the amyloidosis of familial Mediterranean fever. N Engl J Med 314: 1001–1005. 3515182 10.1056/NEJM198604173141601

[2] Sohar E, Gafni J, Pras M, Heller H (1967) Familial Mediterranean fever. A survey of 470 cases and review of the literature. Am J Med 43: 227–253. 5340644 10.1016/0002-9343(67)90167-2

[3] Khachadurian AK, Armenian HK (1974) Familial paroxysmal polyserositis (familial Mediterranean fever); incidence of amyloidosis and mode of inheritance. Birth Defects Orig Artic Ser 10: 62–66. 4470910

[4] Rogers D B, Shohat M, Petersen GM, Bickal J, Congleton J, et al (1989) Familial Mediterranean fever in Armenians: autosomal recessive inheritance with high gene frequency. Am J Med Genet 34: 168–172. 2816993 10.1002/ajmg.1320340206

[5] The International FMF Consortium (1997) Ancient missense mutations in a new member of the RoRet gene family are likely to cause familial Mediterranean fever. Cell 90: 797–807. 9288758 10.1016/s0092-8674(00)80539-5

[6] The French FMF Consortium (1997) A candidate gene for familial Mediterranean fever. Nat Genet 17: 25–31. 9288094 10.1038/ng0997-25

[7] Centola M, Wood G, Frucht DM, Galon J, Aringer M, et al (2000) The gene for familial Mediterranean fever, MEFV, is expressed in early leukocyte development and is regulated in response to inflammatory mediators. Blood 95: 3223–3231. 10807793

[8] Diaz A, Hu C, Kastner DL, Schaner P, Reginato AM, et al (2004) Lipopolysaccharide-induced expression of multiple alternatively spliced MEFV transcripts in human synovial fibroblasts: a prominent splice isoform lacks the C-terminal domain that is highly mutated in familial Mediterranean fever. Arthritis Rheum 50: 3679–3689. 15529356 10.1002/art.20600

[9] Touitou I (2001) The spectrum of Familial Mediterranean Fever (FMF) mutations. Eur J Hum Genet 9: 473–483. 11464238 10.1038/sj.ejhg.5200658

[10] Bernot A, da Silva C, Petit JL, Cruaud C, Caloustian C, et al (1998) Non-founder mutations in the MEFV gene establish this gene as the cause of familial Mediterranean fever (FMF). Hum Mol Genet 7: 1317–1325. 9668175 10.1093/hmg/7.8.1317

[11] Cazeneuve C, Sarkisian T, Pecheux C, Dervichian M, Nedelec B, et al (1999) MEFV-Gene analysis in armenian patients with Familial Mediterranean fever: diagnostic value and unfavorable renal prognosis of the M694V homozygous genotype-genetic and therapeutic implications. Am J Hum Genet 65: 88–97. 10364520 10.1086/302459 PMC1378078

[12] Booth DR, Gillmore JD, Lachmann HJ, Booth SE, Bybee A, et al (2000) The genetic basis of autosomal dominant familial Mediterranean fever. Qjm 93: 217–221. 10787449 10.1093/qjmed/93.4.217

[13] Cazeneuve C, Hovannesyan Z, Genevieve D, Hayrapetyan H, Papin S, et al (2003) Familial Mediterranean fever among patients from Karabakh and the diagnostic value of MEFV gene analysis in all classically affected populations. Arthritis Rheum 48: 2324–2331. 12905488 10.1002/art.11102

[14] van Gijn ME, Soler S, de la Chapelle C, Mulder M, Ritorre C, et al (2008) Search for copy number alterations in the MEFV gene using multiplex ligation probe amplification, experience from three diagnostic centres. Eur J Hum Genet 16: 1404–1406. 18648395 10.1038/ejhg.2008.135

[15] Marek-Yagel D, Berkun Y, Padeh S, Abu A, Reznik-Wolf H, et al (2009) Clinical disease among patients heterozygous for familial Mediterranean fever. Arthritis Rheum 60: 1862–1866. 19479871 10.1002/art.24570

[16] Booty M G, Chae JJ, Masters SL, Remmers EF, Barham B, et al (2009) Familial Mediterranean fever with a single MEFV mutation: where is the second hit? Arthritis Rheum 60: 1851–1861. 19479870 10.1002/art.24569 PMC2753538

[17] Moradian MM, Sarkisian T, Ajrapetyan H, Avanesian N (2010) Genotype-phenotype studies in a large cohort of Armenian patients with familial Mediterranean fever suggest clinical disease with heterozygous MEFV mutations. J Hum Genet 55: 389–393. 20485448 10.1038/jhg.2010.52

[18] Ait-Idir D, Khilan A, Djerdjouri B, El-Shanti H (2011) Spectrum of mutations and carrier frequency of familial Mediterranean fever gene in the Algerian population. Rheumatology (Oxford) 50: 2306–2310. 22019805 10.1093/rheumatology/ker328

[19] Dode C, Pecheux C, Cazeneuve C, Cattan D, Dervichian M, et al (2000) Mutations in the MEFV gene in a large series of patients with a clinical diagnosis of familial Mediterranean fever. Am J Med Genet 92: 241–246. 10842288

[20] Tchernitchko D, Moutereau S, Legendre M, Delahaye A, Cazeneuve C, et al (2005) MEFV analysis is of particularly weak diagnostic value for recurrent fevers in Western European Caucasian patients. Arthritis Rheum 52: 3603–3605. 16255051 10.1002/art.21408

[21] Aldea A, Campistol JM, Arostegui JI, Rius J, Maso M, et al (2004) A severe autosomal-dominant periodic inflammatory disorder with renal AA amyloidosis and colchicine resistance associated to the MEFV H478Y variant in a Spanish kindred: an unusual familial Mediterranean fever phenotype or another MEFV-associated periodic inflammatory disorder? Am J Med Genet A 124A: 67–73. 14679589 10.1002/ajmg.a.20296

[22] Caglar MK, Altugan FS, Ozyurt H, Atasoy HI (2008) Screening of family members of children with Familial Mediterranean Fever: true-autosomal and pseudo-autosomal inheritance. Acta Reumatol Port 33: 415–420. 19107086

[23] Stoffels M, Szperl A, Simon A, Netea MG, Plantinga TS, et al.. (2013) MEFV mutations affecting pyrin amino acid 577 cause autosomal dominant autoinflammatory disease. Ann Rheum Dis ahead of print. 10.1136/annrheumdis-2012-202580 23505238

[24] Livneh A, Langevitz P, Zemer D, Zaks N, Kees S, et al (1997) Criteria for the diagnosis of familial Mediterranean fever. Arthritis Rheum 40: 1879–1885. 9336425 10.1002/art.1780401023

[25] Samuels J, Aksentijevich I, Torosyan Y, Centola M, Deng Z, et al (1998) Familial Mediterranean fever at the millennium. Clinical spectrum, ancient mutations, and a survey of 100 American referrals to the National Institutes of Health. Medicine (Baltimore) 77: 268–297. 9715731 10.1097/00005792-199807000-00005

[26] Ozen S (1999) Vasculopathy, Behcet's syndrome, and familial Mediterranean fever. Curr Opin Rheumatol 11: 393–398. 10503660 10.1097/00002281-199909000-00011

[27] Sarkisian T, Ajrapetian H, Beglarian A, Shahsuvarian G, Egiazarian A (2008) Familial Mediterranean Fever in Armenian population. Georgian Med News 105–111. 18403822

[28] Ozen S, Bakkaloglu A, Yilmaz E, Duzova A, Balci B, et al (2003) Mutations in the gene for familial Mediterranean fever: do they predispose to inflammation? J Rheumatol 30: 2014–2018. 12966608

[29] Lachmann HJ, Sengul B, Yavuzsen TU, Booth DR, Booth S E, et al (2006) Clinical and subclinical inflammation in patients with familial Mediterranean fever and in heterozygous carriers of MEFV mutations. Rheumatology (Oxford) 45: 746–750. 16403826 10.1093/rheumatology/kei279

[30] Kalyoncu M, Acar BC, Cakar N, Bakkaloglu A, Ozturk S, et al (2006) Are carriers for MEFV mutations “healthy”? Clin Exp Rheumatol 24: S120–122. 17067442

[31] Giaglis S, Papadopoulos V, Kambas K, Doumas M, Tsironidou V, et al (2007) MEFV alterations and population genetics analysis in a large cohort of Greek patients with familial Mediterranean fever. Clin Genet 71: 458–467. 17489852 10.1111/j.1399-0004.2007.00789.x

[32] Kone-Paut I, Hentgen V, Guillaume-Czitrom S, Compeyrot-Lacassagne S, Tran TA, et al (2009) The clinical spectrum of 94 patients carrying a single mutated MEFV allele. Rheumatology (Oxford) 48: 840–842. 19465590 10.1093/rheumatology/kep121

[33] Hentgen V, Grateau G, Stankovic-Stojanovic K, Amselem S, Jeru I (2013) Familial Mediterranean fever in heterozygotes: Are we able to accurately diagnose the disease in very young children? Arthritis Rheum ahead of print. 10.1002/art.37935 23508419

[34] Federici S, Calcagno G, Finetti M, Gallizzi R, Meini A, et al (2012) Clinical impact of MEFV mutations in children with periodic fever in a prevalent western European Caucasian population. Ann Rheum Dis 71: 1961–1965. 22580583 10.1136/annrheumdis-2011-200977

[35] Touitou I (2013) Inheritance of autoinflammatory diseases: shifting paradigms and nomenclature. J Med Genet ahead of print. 10.1136/jmedgenet-2013-101577 23536687

[36] Manolio TA, Collins FS, Cox NJ, Goldstein DB, Hindorff LA, et al (2009) Finding the missing heritability of complex diseases. Nature 461: 747–753. 19812666 10.1038/nature08494 PMC2831613

[37] Yilmaz E, Ozen S, Balci B, Duzova A, Topaloglu R, et al (2001) Mutation frequency of Familial Mediterranean Fever and evidence for a high carrier rate in the Turkish population. Eur J Hum Genet 9: 553–555. 11464248 10.1038/sj.ejhg.5200674

[38] Stoffman N, Magal N, Shohat T, Lotan R, Koman S, et al (2000) Higher than expected carrier rates for familial Mediterranean fever in various Jewish ethnic groups. Eur J Hum Genet 8: 307–310. 10854115 10.1038/sj.ejhg.5200446

[39] Al-Alami JR, Tayeh MK, Najib DA, Abu-Rubaiha ZA, Majeed HA, et al (2003) Familial Mediterranean fever mutation frequencies and carrier rates among a mixed Arabic population. Saudi Med J 24: 1055–1059. 14578967

